# Response: Commentary: Predictors of Colorectal Cancer Screening in Two Underserved U.S. Populations: A Parallel Analysis

**DOI:** 10.3389/fonc.2020.00686

**Published:** 2020-04-30

**Authors:** Sarah A. Reisinger, Brittany M. Bernardo, Amy L. Gross, Gregory Young, Ryan Baltic, William J. Blot, Electra D. Paskett

**Affiliations:** ^1^Comprehensive Cancer Center, The Ohio State University, Columbus, OH, United States; ^2^Prevention and Health Promotion Administration, Maryland Department of Health, Baltimore, MD, United States; ^3^Center for Biostatistics, The Ohio State University, Columbus, OH, United States; ^4^Vanderbilt Institute for Clinical and Translational Research, International Epidemiology Field Station, Vanderbilt University Medical Center, Nashville, TN, United States; ^5^Division of Epidemiology, Department of Medicine, School of Medicine, Vanderbilt University, Nashville, TN, United States; ^6^Division of Cancer Prevention and Control, Department of Internal Medicine, College of Medicine, The Ohio State University, Columbus, OH, United States; ^7^Division of Epidemiology, College of Public Health, The Ohio State University, Columbus, OH, United States

**Keywords:** guideline colorectal cancer screening, underserved population, neighborhood deprivation, guideline screening, correlates of screening

In responding to the commentary on our article by Johnson et al., we would like to thank the authors for their effort to highlight the need for additional research in this field. While we agree diet, health beliefs, and other risk factors are certainly important factors and potential contributors to colorectal cancer (CRC) screening adherence, our parallel analysis of two separate, existing cohorts (Ohio Appalachia and the Southern Community Cohort Study) was not designed to capture many of these factors. The data common to both cohorts were specific to the original scope of those studies and were limited to individual and neighborhood characteristics as well as geographic region. We also agree there is a possibility that biological differences in CRC severity may be exacerbated by race/ethnicity, education, obesity, lifestyle/behavior, and lead to increased mortality. We would like to note that our findings of lower CRC screening rates associated with lower area-level and individual socioeconomic status likely support this given the reciprocal relationship these factors have with one's environment. Finally, while our study examined predictors associated with screening adherence within hotspots (areas with higher CRC mortality rates) and we suggested that this information could be used to inform future interventions, a comprehensive study examining both hotspot and non-hotspot areas would be informative. Furthermore, novel investigations such as comparing gut microbiota as suggested by Johnson et al., are imperative in driving future research and key to creating a comprehensive strategy to reduce CRC mortality.

## Author Contributions

SR: conceptualization and writing-original draft preparation. BB, AG, GY, RB, and WB: review and editing. EP: conceptualization, review and editing.

## Conflict of Interest

The authors declare that the research was conducted in the absence of any commercial or financial relationships that could be construed as a potential conflict of interest.

